# Momentary dynamics of inner speech varieties, auditory verbal hallucinations, and affect in schizophrenia spectrum disorders: an experience sampling study

**DOI:** 10.1017/S0033291725102985

**Published:** 2026-01-12

**Authors:** Lawrence Kin-hei Chung, Thomas J. Whitford, Anson Kai Chun Chau, Sandra Sau-man Chan, George Heung-chuen Chong, Suzanne Ho-wai So

**Affiliations:** 1 Department of Psychology, The Chinese University of Hong Kong, Hong Kong SAR, China; 2 School of Psychology, University of New South Wales (UNSW Sydney), Sydney, Australia; 3 Department of Psychiatry, The University of Hong Kong, Hong Kong SAR, China; 4 Department of Psychiatry, The Chinese University of Hong Kong, Hong Kong SAR, China; 5 Clinical Psychology Service, Kwai Chung Hospital, Hong Kong SAR, China

**Keywords:** covert speech, ecological momentary assessment, emotion, psychosis, voices

## Abstract

**Background:**

Auditory verbal hallucinations (AVH) in schizophrenia spectrum disorders (SSDs) may arise from misattributed inner speech. However, it is unclear if inner speech frequency and phenomenology differ in schizophrenia-spectrum voice-hearers compared with healthy individuals, and how different inner speech varieties relate to AVH and affect. Using experience sampling methodology (ESM), this study examined the moment-to-moment dynamics between inner speech varieties, AVH, and affect.

**Methods:**

Participants completed 6 days of ESM on an electronic device, responding to 10 daily prompts on inner speech varieties (i.e. dialogic, evaluative, other people, condensed, and positive), AVH, and affect. Responses from 32 individuals with SSDs with current AVH (‘SSD’) and 34 healthy controls (‘HC’) were analyzed using linear mixed modeling.

**Results:**

SSD reported significantly more inner speech moments and higher momentary intensity of evaluative, other people, condensed, and positive inner speech compared with HC, but not for dialogic inner speech. Within SSD, higher momentary intensities of dialogic, evaluative, other people, and condensed inner speech were associated with higher AVH levels. Momentary negative affect (NA) moderated the association between evaluative inner speech and AVH, with a stronger association at higher NA levels.

**Conclusions:**

SSDs with current AVH experience more frequent inner speech and exhibit a distinct phenomenological profile compared with healthy individuals. Several inner speech varieties are associated with AVH severity momentarily, supporting the hypothesis that inner speech contributes to AVH at the phenomenological level. This study highlights the emotional state as an important moderator of the inner speech–AVH relationship and as a potential therapeutic target.

## Introduction

There is growing recognition of the heterogeneous nature of auditory verbal hallucinations (AVH) – hearing voices without any external stimuli – which have long been considered a cardinal symptom in schizophrenia spectrum disorders (SSDs). Large-scale studies have reported diverse phenomenological characteristics of AVH in people with SSDs, which differed in features such as linguistic complexity, acoustic properties, location, identity, and personal pronouns (Larøi, 2006; Nayani & David, 1996; Plaze et al., 2011; Stephane, Thuras, Nasrallah, & Georgopoulos, 2003). A formative cluster analysis of auditory hallucinations based on their content and themes has identified four different subtypes, namely those that involved commands and running commentary, that sounded like one’s own thought, that were replays of memory, and nonverbal sounds (McCarthy-Jones et al., 2014). Each subtype may map onto distinct neurocognitive mechanisms and require different etiological accounts (Jones, 2010; McCarthy-Jones et al., 2014). For AVH, one prominent model is the inner speech theory, which suggests that some forms of AVH, particularly those that are free-flowing and resemble the verbal mentation typical of inner speech, are a case of misattribution of one’s inner speech to an external source (Allen, Aleman, & McGuire, 2007; Fernyhough, 2004; Jones & Fernyhough, 2007), although the precise mechanistic instantiation remains a matter of debate (Fletcher & Frith, 2009; Moseley, Fernyhough, & Ellison, 2013; Whitford, 2019; Wilkinson & Fernyhough, 2017).

While these neurocognitive accounts provide a potential framework to explain *how* AVH may be generated, they do not address *why* inner speech is readily attributed externally (rather than internally) to form AVH (Fernyhough, 2004). To this end, a developmental approach to inner speech has been proposed (Fernyhough, 1996). Specifically, inner speech under Vygotsky’s view (Vygotsky, 1987) is conceptualized as the end product of a gradual internalization of external dialogue that a child engages with her caretakers, which moves from explicit exchanges with a social target (i.e. conversation) and later on with oneself (i.e. private speech), to fully internalized utterances (i.e. inner speech) that ultimately reduce to a more condensed form, though people can flexibly move between these forms (Fernyhough, 2004; Fernyhough & Borghi, 2023). An upshot of this approach is that inner speech, even in its most abstract form, is “irreducibly dialogic” (Jones & Fernyhough, 2007). In other words, the inherently social nature of inner speech, combined with a faulty self-monitoring system, may heighten the likelihood of external attribution. This could provide one way to explain why AVH often contain back-and-forth qualities in the form of multiple voices, but it remains to be seen how it can be integrated with other frameworks (cf. Kapur, 2003; Morrison, 2001).

It remains unclear whether the frequency and phenomenology of inner speech are different in voice-hearers with SSDs compared with healthy individuals. The re-expansion model of AVH proposed by Fernyhough (2004), which is derived from Vygotsky’s view (Vygotsky, 1987), suggests that while voice-hearers would enjoy a normal *quantity* of inner speech, AVH occur when they misinterpret the normatively re-expanded inner speech as alien under conditions of stress. Indeed, an early study using unstructured interviews found no differences in the quantity and form of inner speech between individuals with SSDs and healthy individuals (Langdon, Jones, Connaughton, & Fernyhough, 2009). However, subsequent studies examining the *quality* of inner speech found mixed results using a psychometrically validated instrument, the Varieties of Inner Speech Questionnaire (VISQ), which, in its revised version, categorized the phenomenological qualities of inner speech into five varieties: dialogic (inner speech that occurs as a back-and-forth conversation), evaluative/critical, other people (inner speech in others’ voices), condensed (abbreviated sentences), and positive/regulatory (Alderson-Day et al., 2018; McCarthy-Jones & Fernyhough, 2011). Specifically, while de Sousa et al. (2016) found that individuals with SSDs endorsed more other people and condensed inner speech than healthy individuals, Rosen et al. (2018) found higher levels of all varieties of inner speech in people with psychosis, except for condensed inner speech. Evaluative and other people inner speech were found to be associated with the severity of hallucinations (de Sousa et al., 2016; Rosen et al., 2018), and higher dialogic inner speech was recently found to be correlated with more severe hallucinations (Mahfoud et al., 2023).

The reliance on participants’ retrospective estimation of inner speech experiences may lead to biases (Heavey & Hurlburt, 2008; Hurlburt, Alderson-Day, Kühn, & Fernyhough, 2016; Hurlburt, Heavey, & Kelsey, 2013). One way to circumvent such issues is to employ experience sampling methodology (ESM) to examine the presence and relations of inner speech, AVH, and affect moment-by-moment, given that these experiences fluctuate across time. As a structured diary in the daily living environment, ESM has been proven to be a feasible and reliable tool to capture subjective momentary experiences like AVH and affect in people with SSDs (Bell et al., 2023; Myin-Germeys et al., 2018; Oorschot, Kwapil, Delespaul, & Myin-Germeys, 2009; So et al., 2020).

The present study aimed to examine the frequency and phenomenological qualities of inner speech based on the 26-item VISQ – Revised (VISQ-R) in individuals with SSDs and current AVH (‘SSD’) and healthy controls (‘HC’) using ESM. As previous cross-sectional studies reported higher levels of distinct varieties of inner speech in people with SSDs compared with healthy participants (de Sousa et al., 2016; Rosen et al., 2018), we hypothesized that SSD would report higher momentary intensity of various inner speech varieties than HC, but the frequency of inner speech moments would be similar between the two groups. Within the SSD participants, based on the established theoretical associations between inner speech and AVH, we hypothesized that the momentary intensity of inner speech varieties would be associated with the momentary intensity of AVH. To evaluate the assertion of the re-expansion model of AVH, where misattributions occur when inner speech is re-expanded under stress (Fernyhough, 2004), we took a simple approach of operationalizing stress as negative affect (NA) and explored the moderating role of momentary affect and baseline negative emotional states in the momentary associations between inner speech varieties and AVH within SSD, given that stress and affect are closely related (Jacobs et al., 2007), and that momentary affective dynamics are disrupted in schizophrenia (So et al., 2023).

## Methods

### Participants

Two groups of adult participants took part in the study: SSD and HC. For the SSD group, inclusion criteria were a diagnosis of an SSD based on the Chinese-bilingual Structured Clinical Interview for Diagnostic and Statistical Manual of Mental Disorders – Fourth Edition (DSM-IV) Axis I Disorders (SCID; So et al., 2003) and an age of 18 years or above. The presence of AVH was defined by scoring 3 or above (from a range of 1–7) on item P3 hallucinatory behavior of the Positive and Negative Syndrome Scale (PANSS; Kay, Fiszbein, & Opler, 1987) in the past week, with follow-up on the Psychotic Symptoms Rating Scales (PSYRATS; Haddock, McCarron, Tarrier, & Faragher, 1999) to confirm that the hallucinations were auditory-verbal in nature. HC participants had no present or past diagnosis of a DSM-IV Axis I disorder (based on SCID). Exclusion criteria for all groups included a history of alcohol or substance dependence within the past year (except nicotine dependence), a history of a neurological illness, drug-induced or organic psychosis, and intellectual disability based on the Wechsler Adult Intelligence Scale – Fourth Edition (Hong Kong) short form (WAIS-IV[HK]; Wechsler, 2008).

SSD participants were outpatients referred by clinicians from public psychiatric services in the New Territories East Cluster and the Kowloon West Cluster of the Hong Kong Hospital Authority. HC participants were recruited through university newsletters and word-of-mouth, and were age- and gender-matched to the SSD group. The study was approved by the Joint Chinese University of Hong Kong-New Territories East Cluster Clinical Research Ethics Committee (2020.477) and the Kowloon West Cluster Research Ethics Committee (KW/EX-21-038(157-03)), and was conducted in accordance with the Declaration of Helsinki. All participants provided written informed consent.

The novelty of our variables (e.g. inner speech and AVH), lack of prior effect size estimates, and the potential for biased estimates with a pilot study (Albers & Lakens, 2018) precluded an a priori power analysis. Thus, we targeted 35 participants per group, drawing on a review of 68 ESM studies in psychosis, which reported a median sample size of 35 (Bell et al., 2023) and recent ESM studies examining within-person effects of AVH in people with SSDs (Fielding-Smith et al., 2020; So et al., 2020). With a maximum of 60 observations per participant (see *ESM procedure*), this sample size should provide adequate power to test the hypotheses.

### Measures

#### Baseline measures

Symptom severity ratings for the SSD participants were obtained by administering the PANSS (Kay, Fiszbein, & Opler, 1987) and the PSYRATS (Haddock, McCarron, Tarrier, & Faragher, 1999) during semi-structured interviews by graduate-level psychologists who were trained and supervised by an experienced clinical psychologist or psychiatrist. The validated Chinese version of the Depression Anxiety Stress Scales – Short Form (DASS-21; Moussa, Lovibond, & Laube, 2001) was used to assess levels of depression, anxiety, and stress. General intelligence was estimated using the WAIS-IV[HK] short form (Wechsler, 2008).

#### ESM measures

All participants were provided with an iPod Touch (fifth generation) preinstalled with a custom-built ESM app. The app emitted a ‘beep’ signal 10 times a day over 6 consecutive days at pseudo-random moments, with each signal at least 30 min apart. The prompts would only occur during participants’ waking hours within a 10–12-h timeframe. This signal-contingent protocol has been shown to lead to satisfactory compliance in our previous ESM studies (So et al., 2020, 2023). Upon each signal, all participants were asked to respond to the same set of ESM items covering inner speech and affect. The SSD group also responded to items relating to AVH. All ESM items were rated on 7-point Likert scales ranging from ‘*Not at all*’ (1) to ‘*Very much*’ (7). Each ESM questionnaire was only accessible for 15 min after the signal emission. Before the actual testing, all participants completed a practice run, during which the distinction between the inner speech and AVH items was emphasized for the SSD group. Specifically, as participants in the SSD group discussed their AVH experiences in detail with the researcher during the assessment interview, they were instructed to only refer to these experiences for the AVH item, and not the inner speech items, particularly when discerning the ‘other people’ inner speech from AVH.

For the inner speech varieties, five questions from the VISQ-R (Alderson-Day et al., 2018) were selected following Alderson and Fernyhough (2015b), wherein the item with the highest factor loading from each factor was selected based on the original VISQ-R factor analysis (Alderson-Day et al., 2018). They were translated into Chinese through a forward-backward translation process by two authors who are proficient in both Chinese and English (LC and SS) and adapted to fit the momentary nature of ESM. They began with the phrase ‘At the time of the alert’, followed by:

1. Were you having a dialogue with yourself in your head? (Dialogic)
2. Were you talking to yourself in a critical way in your head? (Evaluative)
3. Were you having the experience of the voices of other people asking you questions in your head? (Other people)
4. Were you thinking in shortened words compared with your normal, out-loud speech? (Condensed)
5. Were you calming yourself down by talking silently to yourself? (Positive)

An ‘inner speech moment’ (yes/no) was determined by a score >1 (on a 1–7 scale) on any of the five inner speech items in an ESM entry.

For affect, three items (irritated, low, and tense) on NA and three items (cheerful, relaxed, and contented) on positive affect (PA) were included, which have been found to have good internal reliability in our previous ESM studies in people with SSDs (So et al., 2020, 2023). An additional item assessing AVH experience in the SSD group was phrased as follows: ‘At the time of the alert, did you hear voices that other people could not hear?’ (Kimhy et al., 2017; So et al., 2020). An ‘AVH moment’ (yes/no) was determined by a score >1 (on a 1–7 scale) on the AVH item in an ESM entry.

As previous studies have indicated that individuals with SSDs can differentiate between their inner speech and AVH based on the identity of the speaking voice, content, and controllability of the auditory experiences (de Sousa et al., 2016; Hoffman, Varanko, Gilmore, & Mishara, 2008; Langdon, Jones, Connaughton, & Fernyhough, 2009), three adapted items from Hoffman, Varanko, Gilmore, and Mishara (2008) assessing these features were included immediately following the set of inner speech items and the AVH item, respectively, to evaluate the participants’ ability to appraise these subjective experiences moment-to-moment. As these items were designed as branching items, they were re-coded as not applicable when participants’ responses indicated absence of inner speech or AVH moments. They were phrased as follows:

1. Did these inner speeches (or voices) sound the same as your own speaking voice? (Identity)
2. Did these inner speeches (or voices) say things that you would ordinarily think to yourself? (Content)
3. Did you have control over these inner speeches (or voices)? (Controllability)

### Statistical analysis

Statistical analysis was conducted using the GAMLj3 module (version 3.4.2) in jamovi (Gallucci, 2019). First, ESM data were aggregated across moments to compare their mean levels between groups. Next, linear mixed modeling with restricted maximum likelihood estimation was used to account for the multilevel structure of ESM data, where multiple momentary responses (level 1) are nested within individual participants (level 2). All available data were used, and no data imputation was applied to missing data. Outcome variables were entered uncentered, while ESM predictor variables were person-mean centered (within each participant) and baseline predictor variables (i.e. DASS-21 scale scores and PSYRATS auditory hallucinations score) were grand-mean centered (across the entire sample) to disentangle within-person and between-person effects, respectively (Curran & Bauer, 2011; Wang & Maxwell, 2015). In all models, random intercepts were included. Given the moderate sample size, random slopes for the main effects were considered when likelihood ratio tests suggested enhanced model fit, but random slopes for interaction effects were not included to ensure reliable parameter estimation and model parsimony (Lafit et al., 2025). In the case of convergence issues, random slopes that produced the best model fit, as indicated by a lower Akaike Information Criterion, were retained. An independent random-effects covariance structure was specified to permit unique variances for each random effect.

To examine differences in the momentary intensity of inner speech varieties in the two groups, separate models with inner speech varieties (dialogic, evaluative, other people, condensed, and positive) as outcome variables, and Group (HC and SSD) as the predictor, were tested.

Within the SSD group, to examine the momentary associations between inner speech varieties and AVH, as well as the moderating effect of affect and baseline DASS-21 scale scores on these associations, separate models with AVH as outcome variable and the five inner speech varieties, along with their interactions with PA, NA, and baseline DASS-21 scale scores, as predictor variables, were tested. In all models testing for an interaction effect, the main effects were also included.

Exploratory analyses examining the within-moment associations between inner speech varieties and affect, as well as the moderating effect of Group, were performed by testing separate models that included the five inner speech varieties as outcome variables, and PA, NA, and baseline DASS-21 scale scores, along with their interactions with Group, as predictor variables.

## Results

A total of 36 SSD and 37 HC participants were recruited, among whom two were not able to complete the ESM assessment, and five responded to <30% of ESM entries (i.e. at least 18 responses out of 60), and were therefore excluded per previous studies (e.g. So et al., 2020). The final sample consisted of 32 SSD and 34 HC participants, with 1,390 and 1,623 total ESM observations, respectively. The demographic and clinical characteristics of the study participants are summarized in Table 1. There were no differences in age and gender between the two groups (see Table 1).

**Table 1.** Demographic and clinical characteristics of individuals with schizophrenia spectrum disorders and healthy controls

	SSD (*n* = 32)	HC (*n* = 34)	Between-group comparisons
Age (years)	44.5 (14.5)	43.1 (13.6)	*t*(62.96) = 0.42, *p* = .679, *d* = 0.10, 95% CI = [−5.49, 8.37]
Gender, *n* (%)			*χ^2^* (1) = 0.23, *p* = .632, *V* = 0.06
Female	16 (50.0)	15 (44.1)	
Male	16 (50.0)	19 (55.9)	
Education, *n* (%)			*χ^2^* (4) = 18.94, *p* = **.001**, *V* = 0.54
Primary school	2 (6.3)	1 (2.9)	
Secondary school	17 (53.1)	8 (23.5)	
Diploma	8 (25.0)	2 (5.9)	
Bachelor’s degree	4 (12.5)	15 (44.1)	
Master’s degree	1 (3.1)	8 (23.5)	
Employment, *n* (%)			*χ^2^* (1) = 9.47, *p* = **.002**, *V* = 0.38
Yes	16 (50.0)	29 (85.3)	
No	16 (50.0)	5 (14.7)	
Diagnosis (SCID), *n* (%)			
Schizophrenia	28 (87.5)		
Schizoaffective disorder	2 (6.3)		
Schizophreniform disorder	1 (3.1)		
Psychotic disorder NOS	1 (3.1)		
Length of psychiatric care (years)	13.6 (11.7)		
History of hospitalization, *n* (%)			
Yes	26 (81.3)		
No	6 (18.8)		
Number of hospitalizations	3.0 (4.3)		
Antipsychotic medication class, *n* (%)^a^			
Atypical	26 (81.3)		
Atypical + typical	5 (15.6)		
Olanzapine equivalent (mg/day)	20.0 (11.9)^a^		
IQ (WAIS-IV [HK] short form)	94.5 (13.6)	113.5 (10.7)	*t*(58.92) = 6.25, *p* < **.001**, *d* = 1.55, 95% CI = [−25.04, −12.90]
PANSS P3	4.2 (1.0)		
PANSS positive	17.6 (5.2)		
PANSS negative	11.9 (4.1)		
PANSS general psychopathology	26.2 (6.4)		
PANSS total	55.8 (13.0)		
PSYRATS auditory hallucinations	23.0 (6.9)		
PSYRATS delusions	10.6 (7.5)		
PSYRATS total	33.6 (11.7)		
DASS–21 depression	13.3 (11.5)	4.0 (4.6)	*t*(40.10) = 4.29, *p* < **.001**, *d* = 1.07, 95% CI = [4.92, 13.70]
DASS–21 anxiety	15.6 (10.8)	3.4 (3.8)	*t*(38.04) = 6.06, *p* < **.001**, *d* = 1.51, 95% CI = [8.14, 16.29]
DASS–21 stress	17.5 (10.9)	7.5 (6.7)	*t*(50.79) = 4.49, *p* < **.001**, *d* = 1.11, 95% CI = [5.55, 14.51]

*Note*: Mean and standard deviation are presented where appropriate. DASS-21, depression anxiety stress scales – short form; PANSS, positive and negative syndrome scale; PSYRATS, psychotic symptom rating scales. Chi-squared and Welch’s *t*-tests were used as appropriate, and statistically significant comparisons (*p* < .05) are shown in bold.

a *n* = 31.

Table 2 provides the descriptive statistics (i.e. mean levels) of the ESM items in the two groups. Across the 6-day assessment period, the two groups did not differ in the ESM compliance rate (range = 33.0–100%). All SSD participants reported at least one AVH moment, except for one individual, with a total of 952 (69.0%) moments captured. For inner speech moments, all but one participant in each group (a different individual in SSD) reported at least one inner speech moment, totaling 1,181 (85.0%) moments for SSD and 1,036 (63.8%) moments for HC.

**Table 2.** Descriptive statistics of ESM items assessing affect, inner speech, and AVH in individuals with schizophrenia spectrum disorders and healthy controls

	SSD (*n* = 32) mean (SD)	HC (*n* = 34) mean (SD)	Between-group comparisons
Compliance rate	71.8% (18.2%)	79.6% (18.6%)	*t*(63.88) = 1.71, *p* = .093, *d* = 0.42, 95% CI = [−0.01, 0.17]
Inner speech rate	84.7% (27.1%)	64.3% (34.6%)	*t*(61.98) = 2.68, *p* = **.009**, *d* = 0.66, 95% CI = [−0.36, −0.05]
AVH rate	70.3% (39.5%)		
PA	4.4 (1.4)	4.9 (1.0)	*t*(55.65) = 1.50, *p* = .139, *d* = 0.37, 95% CI = [−0.15, 1.05]
NA	3.0 (1.3)	2.3 (0.9)	*t*(53.65) = 2.61, *p* = **.012**, *d* = 0.65, 95% CI = [−1.26, −0.16]
Dialogic IS	2.9 (1.3)	2.7 (1.4)	*t*(63.98) = 0.74, *p* = .465, *d* = 0.18, 95% CI = [−0.89, 0.41]
Evaluative IS	2.5 (1.4)	1.5 (0.6)	*t*(40.49) = 3.85, *p* < **.001**, *d* = 0.96, 95% CI = [−1.54, −0.48]
Other people IS	2.7 (1.3)	1.4 (0.5)	*t*(40.65) = 4.87, *p* < **.001**, *d* = 1.21, 95% CI = [−1.72, −0.71]
Condensed IS	3.0 (1.4)	2.3 (1.2)	*t*(60.73) = 2.05, *p* = **.045**, *d* = 0.51, 95% CI = [−1.28, −0.02]
Positive IS	2.8 (1.4)	2.1 (1.1)	*t*(58.44) = 2.28, *p* = **.026**, *d* = 0.56, 95% CI = [−1.34, −0.09]
Identity (IS)	3.0 (1.3)^a^	4.4 (1.6)^b^	*t*(60.29) = 3.83, *p* < **.001**, *d* = 0.95, 95% CI = [0.65, 2.07]
Content (IS)	3.2 (1.2)^a^	4.6 (1.6)^b^	*t*(59.52) = 4.00, *p* < **.001**, *d* = 1.00, 95% CI = [0.69, 2.08]
Controllability (IS)	3.2 (1.2)^a^	4.7 (1.7)^b^	*t*(57.84) = 4.14, *p* < **.001**, *d* = 1.03, 95% CI = [0.79, 2.26]
AVH	3.1 (1.7)		
Identity (AVH)	2.8 (1.4)^a^		
Content (AVH)	3.2 (1.4)^a^		
Controllability (AVH)	3.3 (1.5)^a^		

*Note*: Inner speech rate refers to the percentage of inner speech moments, determined by a score >1 (on a 1–7 scale) on any of the five inner speech items, across all responded moments. AVH rate refers to the percentage of AVH moments, determined by a score >1 (on a 1–7 scale) on the AVH item, across all responded moments. Percentage, mean, and standard deviations are presented where appropriate. IS, inner speech; NA, negative affect; PA, positive affect. Welch’s *t*-tests were used, and statistically significant comparisons (*p* < .05) are shown in bold.

a *n* = 31.

b *n* = 33.

### Group comparisons on inner speech moments and phenomenological varieties of inner speech

For inner speech, SSD reported a significantly higher mean inner speech rate (i.e. number of inner speech moments) than HC, as shown in Table 2. SSD reported significantly higher momentary intensity of evaluative, *B* = 1.01, standard error [SE] = 0.26, *p* < .001, 95% confidence interval [CI] = [0.51, 1.52]; other people, *B* = 1.22, SE = 0.24, *p* < .001, 95% CI = [0.74, 1.70]; condensed, *B* = 0.65, SE = 0.32, *p* = .044, 95% CI = [0.03, 1.27]; and positive inner speech, *B* = 0.71, SE = 0.31, *p* = .025, 95% CI = [0.10, 1.32], compared with HC. Dialogic inner speech showed no significant group difference, *B* = 0.24, SE = 0.33, *p* = .468, 95% CI = [−0.40, 0.87].

As shown in Table 2, SSD reported significantly lower mean levels in the voice identity, content, and controllability items for inner speech (*p* < .05) than HC. Within the SSD group, paired-sample *t*-tests did not find a significant difference in voice identity, *t*(29) = 1.30, *p* = .205, *d* = 0.24, 95% CI = [−0.11, 0.51]; content, *t*(29) = 0.22, *p* = .825, *d* = 0.04, 95% CI = [−0.24, 0.30]; and controllability, *t*(29) = 1.01, *p* = .320, *d* = 0.18, 95% CI = [−0.46, 0.15], between inner speech and AVH.

For affect, SSD reported significantly higher momentary intensity of NA, *B* = 0.71, SE = 0.27, *p* = .010, 95% CI = [0.18, 1.24], but not PA, *B* = −0.45, SE = 0.30, *p* = .135, 95% CI = [−1.03, 0.13], compared with HC.

### Within the SSD group, were the momentary intensities of inner speech varieties related to the momentary intensity of AVH and baseline AVH severity?

As shown in Table 3, higher levels of dialogic, evaluative, other people, and condensed inner speech, but not positive inner speech, significantly predicted a higher level of AVH. In other words, the more intense the subjective experience of (most of) the inner speech varieties, the more severe the AVH experience was, except for positive self-regulatory speech. All the results remained unchanged after adjusting for IQ and education level.

**Table 3.** Momentary associations between inner speech varieties and AVH, and the moderating effect of momentary affect and baseline negative emotional states in individuals with schizophrenia spectrum disorders (*n* = 32)

Outcome	Moderator	Predictor														
		Dialogic IS *(t)*			Evaluative IS *(t)*			Other people IS *(t)*			Condensed IS *(t)*			Positive IS *(t)*		
		*B*	95% CI	*P*-value	*B*	95% CI	*P*-value	*B*	95% CI	*P*-value	*B*	95% CI	*P*-value	*B*	95% CI	*P*-value
AVH *(t)*	–	**0.17**	**0.04, 0.29**	**.015**	**0.22**	**0.11, 0.33**	**.001**	**0.32**	**0.23, 0.41**	**<.001**	**0.24**	**0.09, 0.38**	**.003**	0.10	−0.01, 0.20	.077
	PA *(t)*	**−0.11**	**−0.17, −0.05**	**<.001**	−0.05	−0.13, 0.02	.157	−0.01	−0.07, 0.05	.799	0.00	−0.07, 0.08	.933	0.01	−0.06, 0.08	.805
	NA *(t)*	−0.03	−0.08, 0.03	.381	**0.09**	**0.04, 0.15**	**.001**	0.03	−0.03, 0.09	.285	0.06	−0.01, 0.13	.087	−0.00	−0.07, 0.06	.891
	DASS–21 depression	−0.00	−0.01, 0.01	.868	0.00	−0.01, 0.01	.910	−0.00	−0.01, 0.01	.587	0.00	−0.01, 0.02	.772	0.00	−0.01, 0.01	.440
	DASS–21 anxiety	0.00	−0.01, 0.01	.781	0.00	−0.01, 0.01	.988	−0.00	−0.01, 0.01	.827	0.00	−0.01, 0.02	.761	0.01	−0.00, 0.02	.242
	DASS–21 stress	0.00	−0.01, 0.01	.879	0.00	−0.01, 0.02	.506	−0.00	−0.01, 0.01	.589	−0.00	−0.01, 0.01	.977	0.01	−0.00, 0.02	.207

*Note:* B, unstandardized fixed regression coefficients; 95% CI, 95% confidence intervals; DASS-21, Depression Anxiety Stress Scales – Short Form; IS, inner speech; NA, negative affect; PA, positive affect. *t* denotes ESM measures. Statistically significant results (*p* < .05) are shown in bold.

Moreover, baseline PSYRATS auditory hallucinations score positively predicted momentary intensities of all inner speech varieties (Dialogic IS: *B* = 0.08, SE = 0.03, *p* = .010, 95% CI = [0.02, 0.15]; Evaluative IS: *B* = 0.11, SE = 0.03, *p* = .002, 95% CI = [0.05, 0.17]; Other people IS: *B* = 0.10, SE = 0.03, *p* = .002, 95% CI = [0.05, 0.16]; Condensed IS: *B* = 0.10, SE = 0.03, *p* = .004, 95% CI = [0.04, 0.17]; Positive IS: *B* = 0.09, SE = 0.03, *p* = .010, 95% CI = [0.03, 0.16]).

### Within the SSD group, were the momentary associations between inner speech varieties and AVH moderated by the momentary NA and baseline negative emotional states?

As shown in Table 3, momentary NA significantly moderated the association of evaluative inner speech with AVH, with simple effects revealing a stronger association between evaluative inner speech and AVH at higher NA levels (mean NA + 1 SD: *B* = 0.23, SE = 0.06, *p* < .001, 95% CI = [0.12, 0.34] vs. mean NA - 1 SD: *B* = 0.08, SE = 0.06, *p* = .173, 95% CI = [−0.04, 0.20]). However, baseline DASS-21 scale scores showed no significant moderating effects.

Momentary PA significantly moderated the association of dialogic inner speech with AVH, with simple effects revealing a stronger association between dialogic inner speech and AVH at lower PA levels (mean PA + 1 SD: *B* = 0.06, SE = 0.07, *p* = .367, 95% CI = [−0.08, 0.20] vs. mean PA - 1 SD: *B* = 0.26, SE = 0.07, *p* < .001, 95% CI = [0.12, 0.39]). All the results remained unchanged after adjusting for IQ and education level.

### Were the momentary intensities of inner speech varieties related to affect, and were these associations moderated by Group?

As shown in Table 4, all five inner speech varieties showed significant positive associations with NA, indicating that higher momentary NA predicted more intense inner speech experiences across both groups. The association between positive inner speech and NA was moderated by Group, with simple effects revealing a stronger positive relationship in the HC group (*B* = 0.41, SE = 0.07, *p* < .001, 95% CI = [0.27, 0.55]) than in the SSD group (*B* = 0.17, SE = 0.08, *p* = .044, 95% CI = [0.00, 0.33]).

**Table 4.** Momentary associations between inner speech varieties and affect in individuals with schizophrenia spectrum disorders (*n* = 32) and healthy controls (*n* = 34)

Predictor	Moderator	Outcome														
		Dialogic IS *(t)*			Evaluative IS *(t)*			Other people IS *(t)*			Condensed IS *(t)*			Positive IS *(t)*		
		*B*	95% CI	*P*-value	*B*	95% CI	*P*-value	*B*	95% CI	*P*-value	*B*	95% CI	*P*-value	*B*	95% CI	*P*-value
PA *(t)*	–	**−0.12**	**−0.24, −0.00**	**.046**	**−0.15**	**−0.22, −0.07**	**<.001**	**−0.08**	**−0.15, −0.01**	**.033**	−0.05	−0.14, 0.04	.271	**−0.13**	**−0.23, −0.03**	**.016**
	Group	0.01	−0.22, 0.25	.912	−0.01	−0.17, 0.15	.904	−0.02	−0.17, 0.13	.817	0.10	−0.08, 0.28	.277	**0.22**	**0.02, 0.42**	**.034**
NA *(t)*	–	**0.25**	**0.13, 0.37**	**<.001**	**0.33**	**0.24, 0.41**	**<.001**	**0.22**	**0.13, 0.31**	**<.001**	**0.20**	**0.10, 0.29**	**<.001**	**0.31**	**0.21, 0.42**	**<.001**
	Group	0.08	−0.17, 0.32	.536	0.08	−0.09, 0.25	.356	0.06	−0.12, 0.25	.501	−0.07	−0.26, 0.13	.515	**−0.25**	**−0.46, −0.04**	**.025**
DASS–21 depression	–	**0.06**	**0.03, 0.09**	**<.001**	**0.09**	**0.07, 0.11**	**<.001**	**0.09**	**0.07, 0.11**	**<.001**	**0.06**	**0.03, 0.09**	**<.001**	**0.07**	**0.04, 0.10**	**<.001**
	Group	−0.03	−0.12, 0.07	.582	0.04	−0.02, 0.11	.212	0.03	−0.03, 0.09	.292	−0.04	−0.14, 0.06	.447	−0.02	−0.11, 0.07	.724
DASS–21 anxiety	–	**0.04**	**0.01, 0.07**	**.007**	**0.08**	**0.05, 0.10**	**<.001**	**0.08**	**0.06, 0.10**	**<.001**	**0.05**	**0.02, 0.08**	**.001**	**0.06**	**0.03, 0.09**	**<.001**
	Group	0.04	−0.08, 0.16	.508	**0.09**	**0.01, 0.17**	**.033**	0.05	−0.03, 0.13	.263	0.01	−0.11, 0.13	.935	0.05	−0.06, 0.16	.397
DASS–21 stress	–	**0.06**	**0.04, 0.09**	**<.001**	**0.08**	**0.06, 0.10**	**<.001**	**0.08**	**0.06, 0.10**	**<.001**	**0.06**	**0.03, 0.08**	**<.001**	**0.07**	**0.05, 0.10**	**<.001**
	Group	−0.05	−0.11, 0.02	.198	0.04	−0.01, 0.09	.105	0.03	−0.02, 0.08	.196	−0.03	−0.10, 0.04	.431	−0.01	−0.08, 0.05	.734

*Note*: *B*, unstandardized fixed regression coefficients; 95% CI, 95% confidence intervals; DASS-21, Depression Anxiety Stress Scales – Short Form; IS, inner speech; NA, negative affect; PA, positive affect. *t* denotes ESM measures. Statistically significant results (*p* < .05) are shown in bold.

Furthermore, all five inner speech varieties showed significant positive associations with DASS-21 depression, DASS-21 anxiety, and DASS-21 stress, respectively, indicating that higher baseline negative emotional states predicted more intense inner speech experiences across both groups. The association between evaluative inner speech and DASS-21 anxiety was moderated by Group, with simple effects revealing a stronger positive relationship in the SSD group (*B* = 0.08, SE = 0.01, *p* < .001, 95% CI = [0.05, 0.11]) than in the HC group (*B* = −0.00, SE = 0.04, *p* = .810, 95% CI = [−0.09, 0.07]).

In contrast, dialogic, evaluative, other people, and positive inner speech were negatively associated with PA, indicating that higher PA predicted reduced intensity of these inner speech varieties in both groups. The association between positive inner speech and PA was moderated by Group, with a stronger negative relationship in the HC group (*B* = −0.23, SE = 0.07, *p* = .002, 95% CI = [−0.36, −0.09]) compared with the SSD group (*B* = −0.00, SE = 0.08, *p* = .964, 95% CI = [−0.16, 0.15]).

## Discussion

This study used ESM to investigate the momentary dynamics of inner speech, AVH, and affect in individuals with SSDs with current AVH (‘SSD’) and non-voice-hearing healthy controls (‘HC’). Using the ESM-adapted VISQ-R (Alderson-Day et al., 2018), our findings reveal elevated inner speech moments and momentary intensity of inner speech varieties in people with SSDs compared with healthy participants. Within the SSD group, momentary inner speech varieties were associated with AVH, and the association between evaluative inner speech and AVH was amplified by momentary NA, underscoring the potential role of NA in the inner speech-AVH relationship.

### Momentary varieties of inner speech

Participants with SSDs reported significantly higher levels of evaluative, other people, condensed, and positive inner speech compared with HC, with no difference in dialogic inner speech. These results align with questionnaire-based studies that showed elevated inner speech varieties in people with psychosis (de Sousa et al., 2016; Rosen et al., 2018), although the specific varieties differed. These results also contrast with Langdon, Jones, Connaughton, and Fernyhough (2009), which found no differences using unstructured interviews. There are at least two reasons that could account for these discrepancies: first, our SSD group involved participants who were experiencing AVH, which differs from previous studies using cohorts with a mixed AVH status; second, the ecological validity of ESM may capture fleeting experiences like inner speech that retrospective methods, such as cross-sectional questionnaires, overlook, which rely on participants’ estimate (Heavey & Hurlburt, 2008).

Importantly, a study comparing the responses collected through ESM and baseline VISQ found that the endorsement of inner speech experiences was typically lower for ESM than generalized self-report (Alderson & Fernyhough, 2015b). Therefore, the fact that people with SSDs who were experiencing AVH reported more inner speech moments and higher intensities of inner speech varieties than HC using ESM may indicate a distinct quantitative feature in this population. Indeed, in-depth descriptive methods have revealed rich inner experiences in schizophrenia (Hurlburt, 1990). Testing this hypothesis with a matched-diagnosis control group (i.e. an SSD group who were either not currently hallucinating or had no voice-hearing history) in future studies would confirm whether an enriched inner speech experience is specific to voice-hearing or SSD in general, and shed light on the functional significance of frequent (inner) self-talk, such as to satisfy a social need in response to loneliness (Reichl, Schneider, & Spinath, 2013), which has been found to increase the likelihood of hearing voices (Brederoo et al., 2023). One intriguing possibility is that ‘excessive’ inner speech could strain the dysfunctional self-monitoring system in people with SSDs, as implicated by recent neurophysiological studies (Chung et al., 2025; Whitford et al., 2025), thereby increasing the risk of misattributing inner speech as AVH. Our results suggest that the assumption that people with SSDs who are experiencing AVH have fewer moments of inner speech than healthy counterparts (Fernyhough, 2004; Lysaker & Lysaker, 2005) – because such experiences are externalized as AVH – may need to be reexamined.

### Inner speech and AVH

Within people with SSDs, elevated levels of dialogic, evaluative, other people, and condensed inner speech, but not positive inner speech, were associated with more severe AVH as measured by both momentary ESM and PSYRATS at baseline. These findings extend previous cross-sectional data (de Sousa et al., 2016; Mahfoud et al., 2023; Rosen et al., 2018) by demonstrating the moment-to-moment parallels between several varieties of inner speech and AVH, and suggest that elevated inner speech experiences may be related to AVH specifically. Crucially, AVH are often dominated by derogatory content and appear in the forms of commands or commentary (Larøi et al., 2019; McCarthy-Jones et al., 2014). The fact that momentary associations with AVH were found for inner speech varieties that involve negative content and back-and-forth conversations, but not for those that are affirmative and encouraging, could be taken as evidence at the phenomenological level that some forms of AVH may, in fact, be misattributed dialogic and evaluative inner speech (Jones & Fernyhough, 2007). More interestingly, despite higher levels of positive inner speech in SSD compared with HC participants, no relationship between positive inner speech and AVH was found, suggesting that negative semantics and/or prosody in inner speech may play an important role in the formation of AVH. That is, the functional link between inner speech generation and misattribution might be dependent on the type or content of the internal verbal mentation. It is worth noting that the present study only examined same-moment associations between inner speech and AVH and may therefore not have captured temporal dynamics that unfold over a longer period of time. One possibility is that positive inner speech could be engaged *in response* to AVH as efforts to self-soothe or to cope with the distress, which would be a worthwhile focus for future studies.

To our knowledge, the moderating effect of momentary NA (but not baseline negative emotional states, i.e. DASS-21 scale scores) on the link between AVH and evaluative inner speech provides the first empirical evidence supporting the re-expansion model’s emphasis on momentary stress as a misattribution trigger (Fernyhough, 2004). These findings indicate that transient negative states, rather than sustained states of negative emotions, may heighten the likelihood of perceiving inner speech as alien, and are consistent with the broader role of NA in driving AVH (Peters et al., 2012; So et al., 2020).

The extent to which the misattribution model, primarily developed for AVH, can be generalized to explain hallucinations in other sensory modalities, particularly those that occur simultaneously or sequentially in time – known as multimodal hallucinations – remains unclear (Montagnese et al., 2021). Considering hallucinatory experiences as generated by separable modality-general and modality-specific processes that interact flexibly within a hierarchically organized system might provide a fruitful conceptual framework for addressing this complexity (Fernyhough, 2019).

### Distinguishing inner speech from AVH

Previous studies examining inner speech in people with SSDs have often *assumed* that participants can separate verbal thoughts from AVH (de Sousa et al., 2016; Langdon, Jones, Connaughton, & Fernyhough, 2009; Rosen et al., 2018), but empirical evidence is scarce. One study by Hoffman, Varanko, Gilmore, and Mishara (2008) using an interview-based questionnaire found that the majority of their participants with SSDs reported being able to differentiate the two experiences most of the time, and the distinction was made primarily based on the identity of the voice (i.e. self or non-self), the verbal content, and how much control they could assert over them. Contrary to these findings, we found that people with SSDs endorsed the levels of these characteristics (i.e. how similar the inner speech/voice is to your own voice, how similar the inner speech/voice content is to your ordinary thoughts, and how much control over the inner speech/voice do you have) similarly for inner speech and AVH, and that HC endorsed them at significantly higher levels for inner speech. Our results thus suggest that people with SSDs may experience a graded difference in the subjective clarity of inner speech compared with healthy individuals.

However, far from showing a uniform relationship, the inner speech varieties showed different dynamics with AVH in our data, which indicates that our participants with SSDs experienced and reported inner speech and AVH as distinct phenomena, and are consistent with the assertion that people with SSDs can accurately identify inner speech. It should be noted that Hoffman, Varanko, Gilmore, and Mishara (2008) only asked about the experiential features people with SSDs used to make the distinction, but did not attempt to test them. One possibility is that while people with SSDs generally have the ability to differentiate between inner speech and AVH in retrospect, it is more difficult for them to make unequivocal judgments in real-time across multiple occasions. It is also possible that they use other features, such as loudness and clarity of the verbal mentation that are not captured in the present study, to make the separation (Hoffman, Varanko, Gilmore, & Mishara, 2008). Future research encompassing a larger variety of features will be needed to shed light on this issue.

### Inner speech and affect

Finally, we found that all inner speech varieties were positively associated with NA momentarily across both groups, which suggests that emotional distress amplifies inner speech regardless of clinical status. These results extend previous research showing that evaluative and other people inner speech were positively correlated with the levels of anxiety and depression in nonclinical populations (Alderson-Day et al., 2018; McCarthy-Jones & Fernyhough, 2011). One speculation is that people were engaged in cognitive processes, such as rumination and mind wandering, which primarily present themselves as inner speech (Alderson-Day & Fernyhough, 2015a; Perrone-Bertolotti et al., 2014). Indeed, more dialogic and evaluative inner speech was engaged during induced rumination (Moffatt et al., 2020). Given the established links between these cognitive processes and negative mood (Goldwin & Behar, 2012; Killingsworth & Gilbert, 2010), it is perhaps unsurprising that associations between dialogic and evaluative inner speech and NA were found in the present study. On the other hand, as positive inner speech often serves the function of emotion regulation (Alderson-Day et al., 2018), it is possible that it was engaged as a coping response against NA. However, the significantly weaker association between positive inner speech and NA in people with SSDs compared with the HC participants suggests reduced regulatory effectiveness in people with SSDs (Visser, Esfahlani, Sayama, & Strauss, 2018), which is consistent with the broader deficits in affect regulation reported in psychosis (O’Driscoll, Laing, & Mason, 2014). Future studies employing techniques such as time-lag analysis would help to clarify the directionality and the cause-and-effect relationships between affect and inner speech.

### Limitations

This study has several limitations. First, given the modest sample size, these results should be confirmed in future studies with larger samples, which may allow the detection of milder interaction effects (e.g. group difference in the NA and IS association). Moreover, the PANSS total score of the SSD group indicated they were only ‘mildly ill’ (Leucht et al., 2005). Along with their chronicity and outpatient status, it is unclear whether the results would generalize to all SSDs voice-hearers and those with more severe symptoms. Including individuals with SSDs without current AVH and nonclinical voice-hearers would be particularly valuable for establishing whether the distinct inner speech profile is specific to AVH or characterizes SSDs more broadly (Toh, Moseley, & Fernyhough, 2022). Second, the use of a single-item ESM measure for each inner speech variety may limit reliability. Although this is a recurring challenge for ESM research (Fritz et al., 2024), including multiple items for each inner speech construct may be one way to improve the validity of measuring inner speech experiences through introspection, which remains an accurate tool in understanding mental contents (Corneille & Gawronski, 2024). Third, while participants were explicitly instructed to report on their inner speech, caution should be exercised when interpreting the responses to the ‘other people’ item in voice-hearers, as it taps into the domain of non-self-voices, potentially blurring the boundary with AVH experiences (Rosen et al., 2018). Future studies may wish to modify the VISQ-R for voice-hearing populations or employ methods, such as descriptive experience sampling, which combines ESM with in-depth interviews, to achieve high-fidelity responses (Hurlburt et al., 2022; Hurlburt, Heavey, & Kelsey, 2013).

## Conclusions

In conclusion, the present study found that individuals with SSDs and current AVH experienced more inner speech moments and heightened moment-to-moment levels of inner speech varieties compared with healthy individuals. Several inner speech varieties were positively associated with AVH severity momentarily, and momentary NA moderated the association between evaluative inner speech and AVH, demonstrating the dynamic interactions between inner speech, AVH, and affect. Taken together, these results offer support for the inner speech theory of AVH at the phenomenological level. Our results suggest that evaluative inner speech and negative emotional state may play a central role in the inner speech–AVH relationship. Tailoring interventions with these potential therapeutic targets may pave the way for more effective and personalized treatments for inner speech-based AVH (Smailes et al., 2015).

## References

[r1] Albers, C., & Lakens, D. (2018). When power analyses based on pilot data are biased: Inaccurate effect size estimators and follow-up bias. Journal of Experimental Social Psychology, 74, 187–195. 10.1016/j.jesp.2017.09.004.

[r2] Alderson-Day, B., & Fernyhough, C. (2015b). Relations among questionnaire and experience sampling measures of inner speech: A smartphone app study. Frontiers in Psychology, 6. 10.3389/fpsyg.2015.00517. PMC4410513 25964773

[r3] Alderson-Day, B., & Fernyhough, C. (2015a). Inner speech: Development, cognitive functions, phenomenology, and neurobiology. Psychological Bulletin, 141(5), 931–965. 10.1037/bul0000021. 26011789 PMC4538954

[r5] Alderson-Day, B., Mitrenga, K., Wilkinson, S., McCarthy-Jones, S., & Fernyhough, C. (2018). The varieties of inner speech questionnaire - revised (VISQ-R): Replicating and refining links between inner speech and psychopathology. Consciousness and Cognition, 65, 48–58. 10.1016/j.concog.2018.07.001. 30041067 PMC6204885

[r6] Allen, P., Aleman, A., & McGuire, P. K. (2007). Inner speech models of auditory verbal hallucinations: Evidence from behavioural and neuroimaging studies. International Review of Psychiatry, 19(4), 407–415. 10.1080/09540260701486498. 17671873

[r8] Bell, I. H., Eisner, E., Allan, S., Cartner, S., Torous, J., Bucci, S., & Thomas, N. (2023). Methodological characteristics and feasibility of ecological momentary assessment studies in psychosis: A systematic review and meta-analysis. Schizophrenia Bulletin, 50(2), 238–265. 10.1093/schbul/sbad127. PMC10919779 37606276

[r9] Brederoo, S. G., de Boer, J. N., Linszen, M. M. J., Blom, R. E., Begemann, M. J. H., & Sommer, I. E. C. (2023). Social deafferentation and the relation between loneliness and hallucinations. Schizophrenia Bulletin, 49(Supplement_1), S25–S32. 10.1093/schbul/sbac064. 36840539 PMC9960004

[r10] Chung, L. K.-h., Whitford, T. J., Griffiths, O., Jack, B. N., Le Pelley, M. E., Spencer, K. M., Barreiros, A. R., Harrison, A. W., Han, N. T., Libesman, S., Pearson, D., Elijah, R. B., Godwin, M., Haroutonian, C., Nickerson, A., Chan, S. S.-m., Chong, G. H.-c., Lau, G. K.-w., Wong, Y.-c., … Harris, A. W. F. (2025). Neurophysiological evidence of motor preparation dysfunction during inner speech and its association with auditory verbal hallucinations in schizophrenia spectrum disorders. Schizophrenia Bulletin. 10.1093/schbul/sbaf219. 41391110

[r11] Corneille, O., & Gawronski, B. (2024). Self-reports are better measurement instruments than implicit measures. Nature Reviews Psychology, 3(12), 835–846. 10.1038/s44159-024-00376-z.

[r12] Curran, P. J., & Bauer, D. J. (2011). The disaggregation of within-person and between-person effects in longitudinal models of change. Annual Review of Psychology, 62(1), 583–619. 10.1146/annurev.psych.093008.100356. PMC3059070 19575624

[r13] de Sousa, P., Sellwood, W., Spray, A., Fernyhough, C., & Bentall, R. P. (2016). Inner speech and clarity of self-concept in thought disorder and auditory-verbal hallucinations. The Journal of Nervous and Mental Disease, 204(12), 885–893. 10.1097/nmd.0000000000000584. 27898489 PMC5142361

[r14] Fernyhough, C. (1996). The dialogic mind: A dialogic approach to the higher mental functions. New Ideas in Psychology, 14(1), 47–62. 10.1016/0732-118x(95)00024-b.

[r15] Fernyhough, C. (2004). Alien voices and inner dialogue: Towards a developmental account of auditory verbal hallucinations. New Ideas in Psychology, 22(1), 49–68. 10.1016/j.newideapsych.2004.09.001.

[r16] Fernyhough, C. (2019). Modality-general and modality-specific processes in hallucinations. Psychological Medicine, 49(16), 2639–2645. 10.1017/s0033291719002496. 31530334 PMC6877466

[r17] Fernyhough, C., & Borghi, A. M. (2023). Inner speech as language process and cognitive tool. Trends in Cognitive Sciences, 27(12), 1180–1193. 10.1016/j.tics.2023.08.014. 37770286

[r18] Fielding-Smith, S. F., Greenwood, K. E., Wichers, M., Peters, E., & Hayward, M. (2020). Associations between responses to voices, distress and appraisals during daily life: An ecological validation of the cognitive behavioural model. Psychological Medicine, 1–10. 10.1017/s0033291720002238. 32646525

[r19] Fletcher, P. C., & Frith, C. D. (2009). Perceiving is believing: A Bayesian approach to explaining the positive symptoms of schizophrenia. Nature Reviews Neuroscience, 10(1), 48–58. 10.1038/nrn2536. 19050712

[r20] Fritz, J., Piccirillo, M. L., Cohen, Z. D., Frumkin, M., Kirtley, O., Moeller, J., … Bringmann, L. F. (2024). So you want to do ESM? 10 essential topics for implementing the experience-sampling method. Advances in Methods and Practices in Psychological Science, 7(3), 2512. 10.1177/25152459241267912.

[r21] Gallucci, M. (2019). GAMLj: General analyses for linear models.[jamovi module].

[r22] Goldwin, M., & Behar, E. (2012). Concreteness of idiographic periods of worry and depressive rumination. Cognitive Therapy and Research, 36(6), 840–846. 10.1007/s10608-011-9428-1.

[r23] Haddock, G., McCarron, J., Tarrier, N., & Faragher, E. (1999). Scales to measure dimensions of hallucinations and delusions: The psychotic symptom rating scales (PSYRATS). Psychological Medicine, 29(4), 879–889. 10.1017/s0033291799008661. 10473315

[r24] Heavey, C. L., & Hurlburt, R. T. (2008). The phenomena of inner experience. Consciousness and Cognition, 17(3), 798–810. 10.1016/j.concog.2007.12.006. 18258456

[r25] Hoffman, R. E., Varanko, M., Gilmore, J., & Mishara, A. L. (2008). Experiential features used by patients with schizophrenia to differentiate ‘voices’ from ordinary verbal thought. Psychological Medicine, 38(8), 1167–1176. 10.1017/s0033291707002395. 18047771

[r26] Hurlburt, R. T. (1990). Sampling normal and schizophrenic inner experience. Plenum Press.

[r27] Hurlburt, R. T., Alderson-Day, B., Kühn, S., & Fernyhough, C. (2016). Exploring the ecological validity of thinking on demand: Neural correlates of elicited vs. spontaneously occurring inner speech. PLoS One, 11(2), e0147932. 10.1371/journal.pone.0147932. 26845028 PMC4741522

[r28] Hurlburt, R. T., Heavey, C. L., & Kelsey, J. M. (2013). Toward a phenomenology of inner speaking. Consciousness and Cognition, 22(4), 1477–1494. 10.1016/j.concog.2013.10.003. 24184987

[r29] Hurlburt, R. T., Heavey, C. L., Lapping-Carr, L., Krumm, A. E., Moynihan, S. A., Kaneshiro, C., … Kelsey, J. M. (2022). Measuring the frequency of inner-experience characteristics. Perspectives on Psychological Science, 17(2), 559–571. 10.1177/1745691621990379. 34283671

[r30] Jacobs, N., Myin-Germeys, I., Derom, C., Delespaul, P., van Os, J., & Nicolson, N. A. (2007). A momentary assessment study of the relationship between affective and adrenocortical stress responses in daily life. Biological Psychology, 74(1), 60–66. 10.1016/j.biopsycho.2006.07.002. 16942831

[r31] Jones, S. R. (2010). Do we need multiple models of auditory verbal hallucinations? Examining the phenomenological fit of cognitive and neurological models. Schizophrenia Bulletin, 36(3), 566–575. 10.1093/schbul/sbn129. 18820262 PMC2879699

[r32] Jones, S. R., & Fernyhough, C. (2007). Neural correlates of inner speech and auditory verbal hallucinations: A critical review and theoretical integration. Clinical Psychology Review, 27(2), 140–154. 10.1016/j.cpr.2006.10.001. 17123676

[r33] Kapur, S. (2003). Psychosis as a state of aberrant salience: A framework linking biology, phenomenology, and pharmacology in schizophrenia. American Journal of Psychiatry, 160(1), 13–23. 10.1176/appi.ajp.160.1.13. 12505794

[r34] Kay, S. R., Fiszbein, A., & Opler, L. A. (1987). The positive and negative syndrome scale (PANSS) for schizophrenia. Schizophrenia Bulletin, 13(2), 261–276. 10.1093/schbul/13.2.261. 3616518

[r35] Killingsworth, M. A., & Gilbert, D. T. (2010). A wandering mind is an unhappy mind. Science, 330(6006), 932–932. 10.1126/science.1192439. 21071660

[r36] Kimhy, D., Wall, M. M., Hansen, M. C., Vakhrusheva, J., Choi, C. J., Delespaul, P., … Malaspina, D. (2017). Autonomic regulation and auditory hallucinations in individuals with schizophrenia: An experience sampling study. Schizophrenia Bulletin, 43(4), 754–763. 10.1093/schbul/sbw219. 28177507 PMC5472124

[r37] Lafit, G., Revol, J., Cloos, L., Kuppens, P., & Ceulemans, E. (2025). The effect of different construct operationalizations, study duration, and preprocessing choices on power-based sample size recommendations in intensive longitudinal research. Assessment, 32(2), 206–223. 10.1177/10731911241286868. 39540648

[r38] Langdon, R., Jones, S. R., Connaughton, E., & Fernyhough, C. (2009). The phenomenology of inner speech: Comparison of schizophrenia patients with auditory verbal hallucinations and healthy controls. Psychological Medicine, 39(4), 655–663. 10.1017/s0033291708003978. 18667096

[r39] Larøi, F. (2006). The phenomenological diversity of hallucinations: Some theoretical and clinical implications. Psychologica Belgica, 46(1–2), 163. 10.5334/pb-46-1-2-163.

[r40] Larøi, F., Thomas, N., Aleman, A., Fernyhough, C., Wilkinson, S., Deamer, F., & McCarthy-Jones, S. (2019). The ice in voices: Understanding negative content in auditory-verbal hallucinations. Clinical Psychology Review, 67, 1–10. 10.1016/j.cpr.2018.11.001. 30553563

[r41] Leucht, S., Kane, J. M., Kissling, W., Hamann, J., Etschel, E., & Engel, R. R. (2005). What does the PANSS mean? Schizophrenia Research, 79(2), 231–238. 10.1016/j.schres.2005.04.008. 15982856

[r42] Lysaker, J., & Lysaker, P. (2005). Being interrupted: The self and schizophrenia. The Journal of Speculative Philosophy, 19(1), 1–21. 10.1353/jsp.2005.0001.

[r43] Mahfoud, D., Hallit, S., Haddad, C., Fekih-Romdhane, F., & Haddad, G. (2023). The moderating effect of cognitive impairment on the relationship between inner speech and auditory verbal hallucinations among chronic patients with schizophrenia. BMC Psychiatry, 23(1), 431. 10.1186/s12888-023-04940-4. 37316820 PMC10265809

[r44] McCarthy-Jones, S., & Fernyhough, C. (2011). The varieties of inner speech: Links between quality of inner speech and psychopathological variables in a sample of young adults. Consciousness and Cognition, 20(4), 1586–1593. 10.1016/j.concog.2011.08.005. 21880511

[r45] McCarthy-Jones, S., Thomas, N., Strauss, C., Dodgson, G., Jones, N., Woods, A., … Sommer, I. E. (2014). Better than mermaids and stray dogs? Subtyping auditory verbal hallucinations and its implications for research and practice. Schizophrenia Bulletin, 40(Suppl_4), S275–S284. 10.1093/schbul/sbu018. 24936087 PMC4141311

[r46] McCarthy-Jones, S., Trauer, T., Mackinnon, A., Sims, E., Thomas, N., & Copolov, D. L. (2014). A new phenomenological survey of auditory hallucinations: Evidence for subtypes and implications for theory and practice. Schizophrenia Bulletin, 40(1), 231–235. 10.1093/schbul/sbs156. 23267192 PMC3885292

[r47] Moffatt, J., Mitrenga, K. J., Alderson-Day, B., Moseley, P., & Fernyhough, C. (2020). Inner experience differs in rumination and distraction without a change in electromyographical correlates of inner speech. PLoS One, 15(9), e0238920. 10.1371/journal.pone.0238920. 32925961 PMC7489561

[r48] Montagnese, M., Leptourgos, P., Fernyhough, C., Waters, F., Larøi, F., Jardri, R., McCarthy-Jones, S., Thomas, N., Dudley, R., Taylor, J.-P., Collerton, D., & Urwyler, P. (2021). A review of multimodal hallucinations: Categorization, assessment, theoretical perspectives, and clinical recommendations. Schizophrenia Bulletin, 47(1), 237–248. 10.1093/schbul/sbaa101. 32772114 PMC7825001

[r49] Morrison, A. P. (2001). The interpretation of intrusions in psychosis: An integrative cognitive approach to hallucinations and delusions. Behavioural and Cognitive Psychotherapy, 29(3), 257–276. 10.1017/S1352465801003010.

[r50] Moseley, P., Fernyhough, C., & Ellison, A. (2013). Auditory verbal hallucinations as atypical inner speech monitoring, and the potential of neurostimulation as a treatment option. Neuroscience and Biobehavioral Reviews, 37(10 Pt 2), 2794–2805. 10.1016/j.neubiorev.2013.10.001. 24125858 PMC3870271

[r51] Moussa, M., Lovibond, P., & Laube, R. (2001). Psychometric properties of a Chinese version of the short depression anxiety stress scales (DASS21). Sydney: Cumberland Hospital.

[r52] Myin-Germeys, I., Kasanova, Z., Vaessen, T., Vachon, H., Kirtley, O., Viechtbauer, W., & Reininghaus, U. (2018). Experience sampling methodology in mental health research: New insights and technical developments. World Psychiatry, 17(2), 123–132. 10.1002/wps.20513. 29856567 PMC5980621

[r53] Nayani, T. H., & David, A. S. (1996). The auditory hallucination: A phenomenological survey. Psychological Medicine, 26(1), 177–189. 10.1017/s003329170003381x. 8643757

[r54] O’Driscoll, C., Laing, J., & Mason, O. (2014). Cognitive emotion regulation strategies, alexithymia and dissociation in schizophrenia, a review and meta-analysis. Clinical Psychology Review, 34(6), 482–495. 10.1016/j.cpr.2014.07.002. 25105273

[r55] Oorschot, M., Kwapil, T., Delespaul, P., & Myin-Germeys, I. (2009). Momentary assessment research in psychosis. Psychological Assessment, 21(4), 498–505. 10.1037/a0017077. 19947784

[r56] Perrone-Bertolotti, M., Rapin, L., Lachaux, J. P., Baciu, M., & Loevenbruck, H. (2014). What is that little voice inside my head? Inner speech phenomenology, its role in cognitive performance, and its relation to self-monitoring. Behavioural Brain Research, 261, 220–239. 10.1016/j.bbr.2013.12.034. 24412278

[r57] Peters, E., Lataster, T., Greenwood, K., Kuipers, E., Scott, J., Williams, S., … Myin-Germeys, I. (2012). Appraisals, psychotic symptoms and affect in daily life. Psychological Medicine, 42(05), 1013–1023. 10.1017/s0033291711001802. 21910936

[r58] Plaze, M., Paillere-Martinot, M. L., Penttila, J., Januel, D., De Beaurepaire, R., Bellivier, F., … Cachia, A. (2011). Where do auditory hallucinations come from?" – A brain morphometry study of schizophrenia patients with inner or outer space hallucinations. Schizophrenia Bulletin, 37(1), 212–221. 10.1093/schbul/sbp081. 19666833 PMC3004180

[r59] Reichl, C., Schneider, J. F., & Spinath, F. M. (2013). Relation of self-talk frequency to loneliness, need to belong, and health in German adults. Personality and Individual Differences, 54(2), 241–245. 10.1016/j.paid.2012.09.003.

[r60] Rosen, C., McCarthy-Jones, S., Chase, K. A., Humpston, C. S., Melbourne, J. K., Kling, L., & Sharma, R. P. (2018). The tangled roots of inner speech, voices and delusions. Psychiatry Research, 264, 281–289. 10.1016/j.psychres.2018.04.022. 29660570 PMC5972053

[r61] Smailes, D., Alderson-Day, B., Fernyhough, C., McCarthy-Jones, S., & Dodgson, G. (2015). Tailoring cognitive behavioral therapy to subtypes of voice-hearing. Frontiers in Psychology, 6. 10.3389/fpsyg.2015.01933. PMC4685120 26733919

[r62] So, E., Kam, I., Leung, C. M., Chung, D., Liu, Z., & Fong, S. (2003). The Chinese-bilingual SCID-I/P project: Stage 1-reliability for mood disorders and schizophrenia. Hong Kong Journal of Psychiatry, 13(1), 7–18.

[r63] So, S. H.-w., Chau, A. K. C., Chung, L. K.-H., Leung, C.-M., Chong, G. H. C., Chang, W. C., … Sommer, I. E. (2023). Moment-to-moment affective dynamics in schizophrenia and bipolar disorder. European Psychiatry, 1–38. 10.1192/j.eurpsy.2023.2438. 37544924 PMC10594258

[r64] So, S. H.-W., Chung, L. K.-H., Tse, C.-Y., Chan, S. S.-M., Chong, G. H.-C., Hung, K. S.-Y., & Sommer, I. E. C. (2020). Moment-to-moment dynamics between auditory verbal hallucinations and negative affect and the role of beliefs about voices. Psychological Medicine, 1–7. 10.1017/s0033291719003611. 31907105

[r65] Stephane, M., Thuras, P., Nasrallah, H., & Georgopoulos, A. P. (2003). The internal structure of the phenomenology of auditory verbal hallucinations. Schizophrenia Research, 61(2–3), 185–193. 10.1016/s0920-9964(03)00013-6. 12729870

[r66] Toh, W. L., Moseley, P., & Fernyhough, C. (2022). Hearing voices as a feature of typical and psychopathological experience. Nature Reviews Psychology, 1(2), 72–86. 10.1038/s44159-021-00013-z.

[r67] Visser, K. F., Esfahlani, F. Z., Sayama, H., & Strauss, G. P. (2018). An ecological momentary assessment evaluation of emotion regulation abnormalities in schizophrenia. Psychological Medicine, 48(14), 2337–2345. 10.1017/S0033291717003865. 29361997

[r68] Vygotsky, L. S. (1987). Thinking and speech. In R. W. Rieber & A. S. Carton (Eds.), The collected works of L. S. Vygotsky (Vol. 1). Plenum.

[r69] Wang, L. P., & Maxwell, S. E. (2015). On disaggregating between-person and within-person effects with longitudinal data using multilevel models. Psychological Methods, 20(1), 63–83. 10.1037/met0000030. 25822206

[r70] Wechsler, D. (2008). Wechsler adult intelligence scale—Fourth edition. Pearson Assessment.

[r71] Whitford, T. J. (2019). Speaking-induced suppression of the auditory cortex in humans and its relevance to schizophrenia. Biological Psychiatry: Cognitive Neuroscience and Neuroimaging, 4(9), 791–804. 10.1016/j.bpsc.2019.05.011. 31399393

[r72] Whitford, T. J., Chung, L. K.-h., Griffiths, O., Jack, B. N., Le Pelley, M. E., Spencer, K. M., Barreiros, A. R., Harrison, A. W., Han, N. T., Libesman, S., Pearson, D., Elijah, R. B., Godwin, M., Haroutonian, C., Nickerson, A., Chan, S. S.-m., Chong, G. H.-c., Lau, G. K.-w., Wong, Y.-c., … So, S. H.-w. (2025). Corollary discharge dysfunction to inner speech and its relationship to auditory verbal hallucinations in patients with schizophrenia spectrum disorders. Schizophrenia Bulletin. 10.1093/schbul/sbaf167. PMC13645647 41116234

[r73] Wilkinson, S., & Fernyhough, C. (2017). Auditory verbal hallucinations and inner speech: A predictive processing perspective. In Z. Radman (Ed.), Before consciousness: In search of the fundamentals of mind (pp. 285–304). Imprint Academic. 28825786

